# DUSP6 Deficiency Attenuates Neurodegeneration after Global Cerebral Ischemia

**DOI:** 10.3390/ijms24097690

**Published:** 2023-04-22

**Authors:** Yi-Chinn Weng, Yu-Ting Huang, I-Chen Chiang, Huai-Chia Chuang, Tsong-Hai Lee, Tse-Hua Tan, Wen-Hai Chou

**Affiliations:** 1 Center for Neuropsychiatric Research, National Health Research Institutes, Miaoli County 35053, Taiwan; 2 Immunology Research Center, National Health Research Institutes, Zhunan, Miaoli County 35053, Taiwan; 3 Stroke Center and Department of Neurology, Linkou Chang Gung Memorial Hospital and College of Medicine, Chang Gung University, Taoyuan 33305, Taiwan

**Keywords:** DUSP6, MKP3, global cerebral ischemia, neurodegeneration, ERK1/2

## Abstract

Transient global cerebral ischemia (tGCI) resulting from cardiac arrest causes selective neurodegeneration in hippocampal CA1 neurons. Although the effect is clear, the underlying mechanisms directing this process remain unclear. Previous studies have shown that phosphorylation of Erk1/2 promotes cell survival in response to tGCI. DUSP6 (also named MKP3) serves as a cytosolic phosphatase that dephosphorylates Erk1/2, but the role of DUSP6 in tGCI has not been characterized. We found that DUSP6 was specifically induced in the cytoplasm of hippocampal CA1 neurons 4 to 24 h after tGCI. DUSP6-deficient mice showed normal spatial memory acquisition and retention in the Barnes maze. Impairment of spatial memory acquisition and retention after tGCI was attenuated in DUSP6-deficient mice. Neurodegeneration after tGCI, revealed by Fluoro-Jade C and H&E staining, was reduced in the hippocampus of DUSP6-deficient mice and DUSP6 deficiency enhanced the phosphorylation and nuclear translocation of Erk1/2 in the hippocampal CA1 region. These data support the role of DUSP6 as a negative regulator of Erk1/2 signaling and indicate the potential of DUSP6 inhibition as a novel therapeutic strategy to treat neurodegeneration after tGCI.

## 1. Introduction

Cardiac arrest is a major cause of mortality and disability worldwide, afflicting more than 550,000 people in the United States and 10,000 people in Taiwan each year [1,2]. Cardiac arrest is a sudden loss of heart beat and cardiac mechanical activity, resulting in severe reduction of blood flow and global cerebral ischemia [3,4]. Cardiopulmonary resuscitation (CPR) is the most urgent lifesaving intervention carried out to rescue patients with cardiac arrest [3,4]. Targeted temperature management, also known as therapeutic hypothermia, is used to lower head temperature to the range of 32–34 °C [1,5]. Targeted temperature management was introduced about 10 years ago and continues to be the leading therapy for cardiac arrest. Despite the expanded use of CPR and targeted temperature management, only 11.5% of cardiac arrest patients survive until hospital discharge [1]. Therefore, new therapeutic strategies for cardiac arrest patients are urgently needed.

The hippocampus is one of the brain regions most vulnerable to global cerebral ischemia after cardiac arrest [6,7,8]. Transient global cerebral ischemia (tGCI) induces selective neurodegeneration in the CA1 region of the hippocampus at 3 days after reperfusion, but leaves the dentate gyrus (DG) region intact [7]. Extracellular signal-regulated kinase 1/2 (ERK 1/2), a member of the mitogen-activated protein kinase (MAPK) family, regulates a wide variety of cellular activities [8,9,10,11]. Erk1/2 is transiently phosphorylated and activated in the surviving DG region 30 min after tGCI, but not in the vulnerable CA1 region [7]. Dual roles of ERK1/2 in cerebral ischemia have been demonstrated but, in general, ERK1/2 activation provides neuroprotection after an ischemic event [8,9,10,11].

Dual-specific phosphatase-6 (DUSP6), also known as mitogen-activated protein kinase phosphatase-3 (MKP3), has been characterized as a negative feedback regulator of ERK1/2 signaling [12,13]. DUSP6 belongs to a family of 25 protein phosphatases that dephosphorylate phosphotyrosine and phosphoserine/phosphothreonine residues within the same substrate [12]. DUSPs are negative and spatiotemporal regulators of MAPK signaling [13], and are classified as typical or atypical based on the presence or absence of an MAPK interacting domain, respectively. Typical DUSPs are further divided into three groups, depending on their subcellular locations: nuclear, cytoplasmic, or dually located. DUSP6 is a cytoplasmic DUSP known to catalyze the removal of phosphate groups from threonine and tyrosine residues on Erk1/2 [12]. Previous studies have identified essential roles of DUSP6 in heart development [14], diet-induced obesity [15,16,17], metabolism [18,19], T cell differentiation [20], and the pluripotency of embryonic stem cells [21,22]. However, the role of DUSP6 in cerebral ischemia remains elusive.

In this study, we investigated the expression and function of DUSP6 in the context of cerebral ischemia. We found that DUSP6 was specifically induced in the cytoplasm of neurons in the vulnerable CA1 region of the hippocampus, but not in the surviving DG region, after tGCI. DUSP6 deficiency enhanced Erk1/2 phosphorylation in the CA1 region and reduced ischemia-induced neurodegeneration after tGCI. These results suggest an important role of DUSP6 in mediating the survival of hippocampal neurons after tGCI and highlight the potential for use of DUSP6 inhibition as a novel therapy to attenuate ischemia-induced neurodegeneration after cardiac arrest.

## 2. Results

### 2.1. Transient Induction of DUSP6 in the Cytoplasm of Hippocampal CA1 Neurons after tGCI

**Figure 1:**
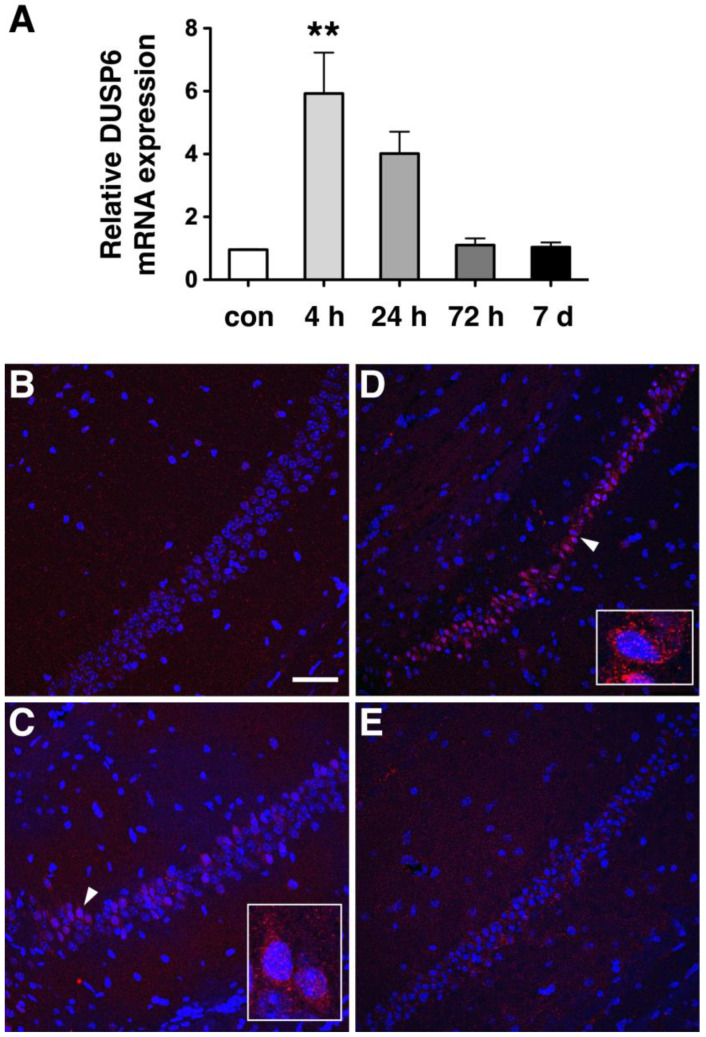
DUSP6 expression was induced in the hippocampus from 4 to 24 h after tGCI. (**A**) Total RNA isolated from the hippocampus of naïve mice (con) and from mice at 4, 24, and 72 h, and 7 days after tGCI, were analyzed by real-time RT-PCR (*n* = 3 per group) using GAPDH mRNA as an internal control. Relative DUSP6 expression in the hippocampus at different time points was compared using one-way ANOVA and Tukey’s post hoc tests. The mRNA levels of DUSP6 at 4 h after tGCI were significantly elevated compared with those of naïve mice (** *p* < 0.01). Expression of DUSP6 protein in the hippocampus of naïve mice (**B**), and at 4 h (**C**), 24 h (**D**), and 48 h (**E**) after tGCI was detected using immunofluorescence staining and confocal microscopy. Nuclei were stained with DAPI (blue). Representative neurons (arrowhead) are amplified in the lower right side of panels C and D. Scale bar, 50 μm.

**Figure 2:**
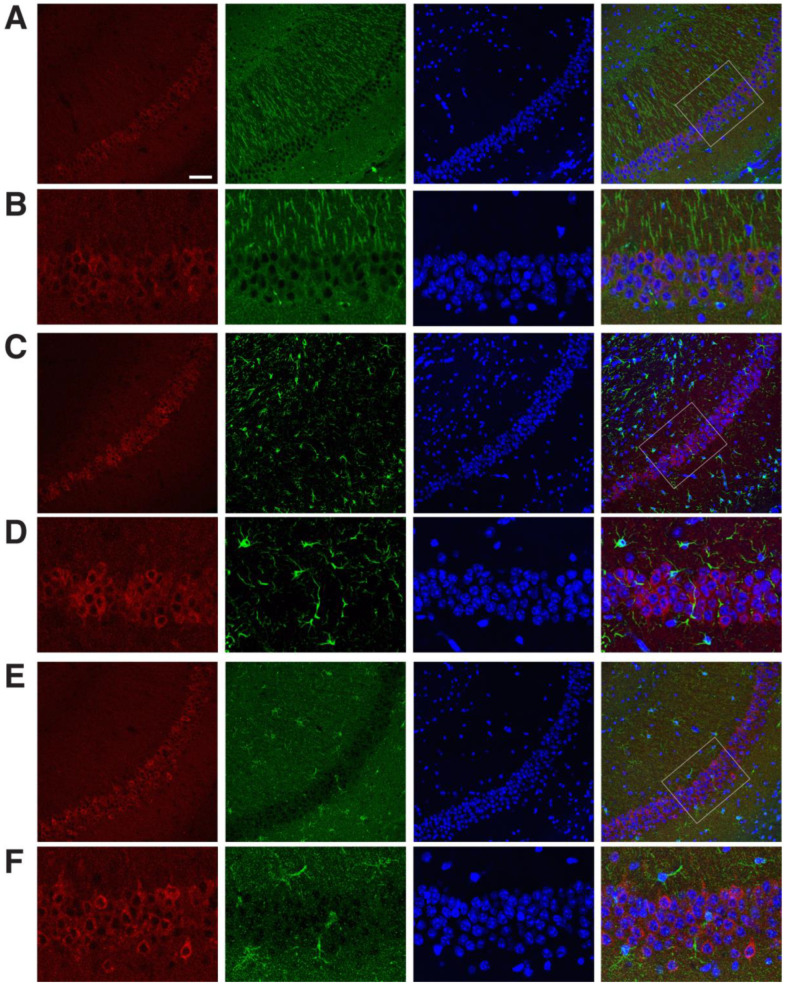
Expression pattern of DUSP6 protein in the hippocampal CA1 region at 24 h after tGCI. (**A**,**B**) Mouse brain sections isolated 24 h after tGCI were labeled with antibodies recognizing DUSP6 (red), MAP2 (green), and DAPI (blue). The merged confocal image shows the expression of DUSP6 protein in the cytoplasm of MAP2-positive neurons in the hippocampal CA1 region. Confocal images of DUSP6-positive cells (red) and GFAP-positive astrocytes (green, **C**,**D**) or Iba1-positive microglia (green, **E**,**F**) in the hippocampal CA1 region 24 h after tGCI demonstrate lack of colocalization. Enlarged views of boxed areas in panel (**A**,**C**,**E**) are shown in panel (**B**,**D**,**F**), respectively. Scale bars, 50 μm.

To assess the expression of DUSP6 in response to tGCI, we occluded bilateral common carotid arteries for 10 min and dissected the hippocampus at 4, 24, and 72 h, and 7 days after reperfusion. Real-time RT–PCR analysis revealed that the levels of DUSP6 mRNA were elevated at 4 and 24 h and returned to the basal level at 72 h and 7 days after tGCI (Figure 1A). To confirm the induction of DUSP6 in the hippocampus, brain sections isolated at 4, 24, and 48 h after tGCI were stained with an antibody recognizing DUSP6 (Figure 1B–E). DUSP6 immunoreactivity was detectable in the hippocampus of naïve mice (Figure 1B) and elevated in the hippocampal CA1 region close to the subiculum as early as 4 h after tGCI (Figure 1C). The number of DUSP6-positive cells in the hippocampus continued to rise at 24 h (Figure 1D) and diminished in the hippocampal CA1 region by 48 h after tGCI (Figure 1E). To determine the types of cells expressing DUSP6, brain sections isolated at 24 h after tGCI were stained with antibodies recognizing DUSP6 and specific markers for neurons (MAP2), astrocytes (GFAP), or microglia (Iba1) (Figure 2). The presence of DUSP6 protein was observed in the cytoplasm of MAP2-positive neurons (Figure 2A,B), while it was not detected in the GFAP-positive astrocytes (Figure 2C,D) or Iba1-positive microglia (Figure 2E,F) in the hippocampal CA1 region at 24 h after tGCI.

### 2.2. DUSP6 Deficiency Reduced the Impairment of Spatial Learning and Memory after tGCI

**Figure 3:**
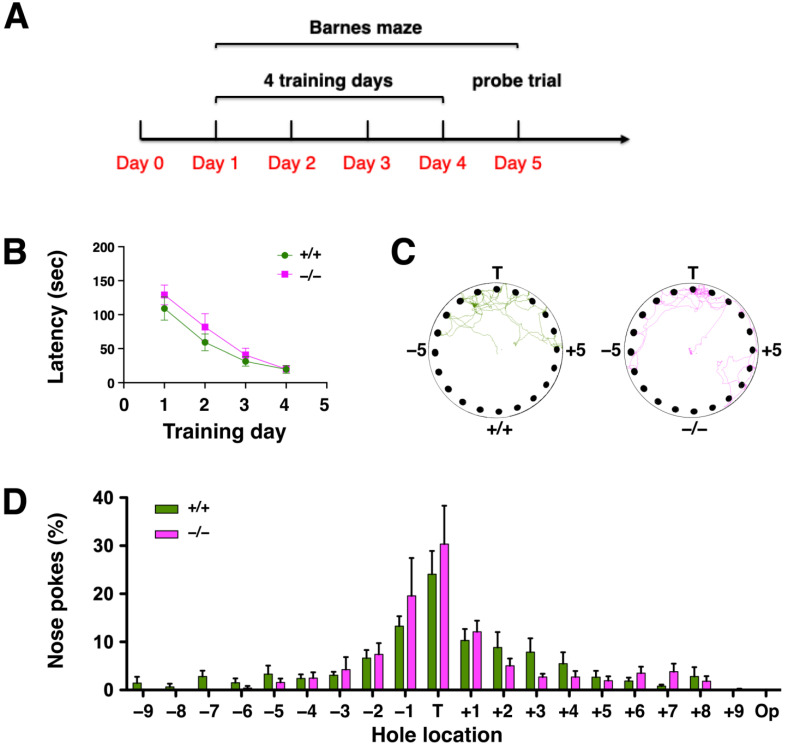
*DUSP6^−/−^* mice displayed normal spatial learning and memory in the Barnes maze. (**A**) Design of the Barnes maze experiment. (**B**) The escape latency (sec) to the target hole during the 4 days of training in the Barnes maze were recorded for naïve *DUSP6^+/+^* (*n* = 8, green circles) and *DUSP6^−/−^* (*n* = 8, pink squares) mice. (**C**) Representative path tracings of naïve *DUSP6^+/+^* and *DUSP6^−/−^* mice during the probe trial on the fifth day of Barnes maze experiment. (**D**) The histogram shows the percentage of nose pokes in each hole relative to overall nose pokes during the probe trial for naïve *DUSP6^+/+^* (*n* = 8, green) and *DUSP6^−/−^* (*n* = 8, pink) mice. Numbers refer to the location of holes adjacent to the target hole (T). Op, Opposite hole.

The functional role of the hippocampus in spatial learning and memory has been established [23,24]. To assess the effect of DUSP6 deficiency on spatial learning and memory, experimentally-naïve *DUSP6^+/+^* and *DUSP6^−/−^* mice were tested using the Barnes maze (Figure 3A). Two-way ANOVA revealed that naïve *DUSP6^+/+^* and *DUSP6^−/−^* mice learned the Barnes maze task with progressively reduced latency (*F*_day_ (3,42) = 43.61, *p* < 0.0001) in locating the target hole across the four training days (Figure 3B). No significant difference was found between naïve *DUSP6^+/+^* and *DUSP6^−/−^* mice in escape latency (*F*_genotype_ (1,14) = 0.96, *p* = 0.3443) (Figure 3B). These results suggest that DUSP6 deficiency did not alter spatial learning and memory function. To assess the retrieval of spatial memory, a probe trial was performed with the escape box removed from the maze on the fifth day of the Barnes maze experiment (Figure 3C,D). The path tracings of naïve *DUSP6^+/+^* and *DUSP6^−/−^* mice during the probe trial were concentrated around the target hole (Figure 3C). The percentage of nose pokes was highest at the target hole and distributed similarly between naïve *DUSP6^+/+^* and *DUSP6^−/−^* mice (Figure 3D). These results suggest that DUSP6 deficiency did not alter memory retrieval function.

**Figure 4:**
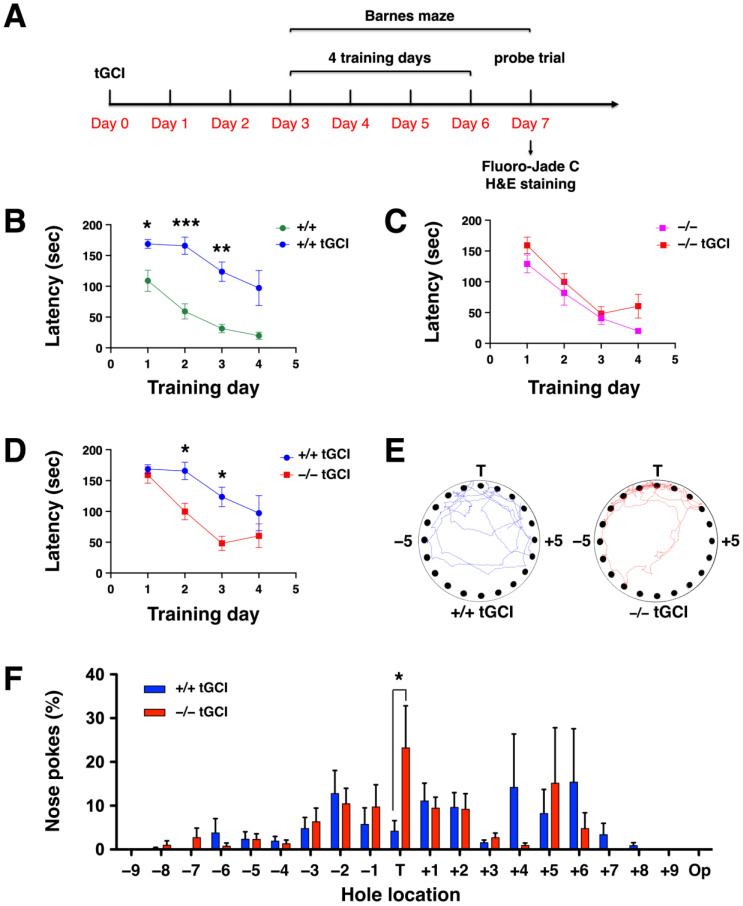
Spatial learning and memory in the Barnes maze was sustained in *DUSP6^−/−^* mice after tGCI. (**A**) Experimental design for the Barnes maze and histological staining following tGCI. (**B**) The escape latency (sec) to the target hole was recorded for naïve *DUSP6^+/+^* mice (*n* = 8, green circles) and *DUSP6^+/+^* mice after tGCI (*n* = 6, blue circles) (* *p* < 0.05, ** *p* < 0.01, *** *p* < 0.001). (**C**) The escape latency (sec) to the target hole was recorded for naïve *DUSP6^−/−^* mice (*n* = 8, pink squares) and *DUSP6^−/−^* mice after tGCI (*n* = 6, red squares). (**D**) The escape latency (sec) to the target hole was recorded for *DUSP6^+/+^* mice after tGCI (*n* = 6, blue circles) and *DUSP6^−/−^* mice after tGCI (*n* = 6, red squares) (* *p* < 0.05). (**E**) Representative path tracings of *DUSP6^+/+^* and *DUSP6^−/−^* mice after tGCI during the probe trial. (**F**) The histogram shows the percentage of nose pokes in each hole relative to overall nose pokes during the probe trial for *DUSP6^+/+^* mice after tGCI (*n* = 6, blue) and *DUSP6^−/−^* mice after tGCI (*n* = 6, red) (* *p* < 0.05). Numbers refer to the location of holes adjacent to the target hole (T). Op, Opposite hole.

To determine the effect of cerebral ischemia on spatial learning and memory, *DUSP6^+/+^* and *DUSP6^−/−^* mice were subjected to tGCI followed by 7 days of recovery (Figure 4A). *DUSP6^+/+^* and *DUSP6^−/−^* mice were trained in the Barnes maze from days 3 to 6 after tGCI. Two-way ANOVA revealed a significant difference in escape latency between naïve *DUSP6^+/+^* and *DUSP6^+/+^* mice after tGCI (*F*_ischemia_ (1,12) = 30.37, *p* = 0.0001) (Figure 4B). Bonferroni’s multiple comparisons test showed that *DUSP6^+/+^* mice after tGCI had significantly greater latency than naïve *DUSP6^+/+^* mice in locating the target hole on training days 1, 2, and 3 (Figure 4B), suggesting that cerebral ischemia disrupted spatial memory acquisition and retention. Interestingly, no significant difference in escape latency was found between naïve *DUSP6^−/−^* mice and *DUSP6^−/−^* mice after tGCI (*F*_ischemia_ (1,12) = 2.79, *p* = 0.1208) (Figure 4C). Moreover, Bonferroni’s multiple comparisons test demonstrated that, after tGCI in both groups, *DUSP6^−/−^* mice had significantly reduced latency compared to *DUSP6^+/+^* mice in locating the target hole on training days 2 and 3 (*F*_genotype_ (1,10) = 8.05, *p* = 0.0176) (Figure 4D). These results suggest that DUSP6 deficiency reduced the impairment of spatial memory acquisition and retention caused by cerebral ischemia. A probe trial was performed to assess the retrieval of spatial memory on day 7 after tGCI (Figure 4E,F). The path tracings of *DUSP6^+/+^* mice after tGCI were dispersed in the upper half of the maze, whereas the tracings of *DUSP6^−/−^* mice after tGCI remained concentrated at the target hole (Figure 4E). The percentage of nose pokes at the target hole was significantly reduced in *DUSP6^+/+^* mice compared to *DUSP6^−/−^* mice after tGCI in both groups (Figure 4F), indicating that DUSP6 deficiency reduced the impairment of memory retrieval after cerebral ischemia. These data suggest that neurodegeneration after tGCI is reduced in *DUSP6^−/−^* mice.

### 2.3. DUSP6 Deficiency Attenuated Neurodegeneration after tGCI

**Figure 5:**
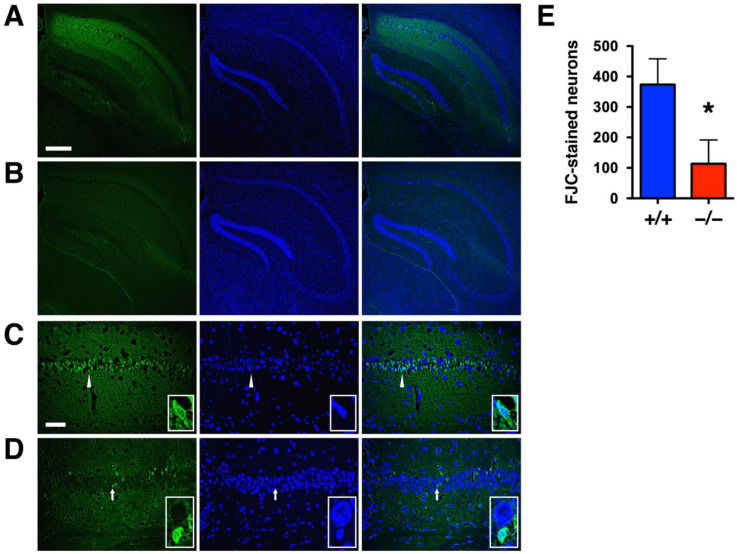
*DUSP6^−/−^* mice showed attenuated neurodegeneration after tGCI. Representative images of Fluoro-Jade C-stained brain sections from *DUSP6^+/+^* mice (**A**) and *DUSP6^−/−^* mice (**B**) subjected to 10 min of tGCI followed by 7 days of recovery. Enlarged views of the hippocampal CA1 region showing greater numbers of Fluoro-Jade C-stained neurons in *DUSP6^+/+^* mice (**C**) than those in *DUSP6^−/−^* mice (**D**) after tGCI. An enlarged image of a degenerating neuron with a shrunken nucleus (arrowhead) from the *DUSP6^+/+^* mouse is included in the lower right corner of each panel of part C. An enlarged image of a healthy neuron with a large round nucleus and a degenerating neuron with shrunken nucleus (arrow) from the *DUSP6^−/−^* mouse is included in the lower right corner of each panel of part D. Scale bars, 250 μm (**A**) and 50 μm (**C**). (**E**) The number of Fluoro-Jade C (FJC)-stained neurons was significantly reduced in *DUSP6^−/−^* mice (*n* = 6 per group) 7 days after tGCI. * *p* < 0.05 compared with *DUSP6^+/+^* mice (two-tailed, unpaired *t*-test).

**Figure 6:**
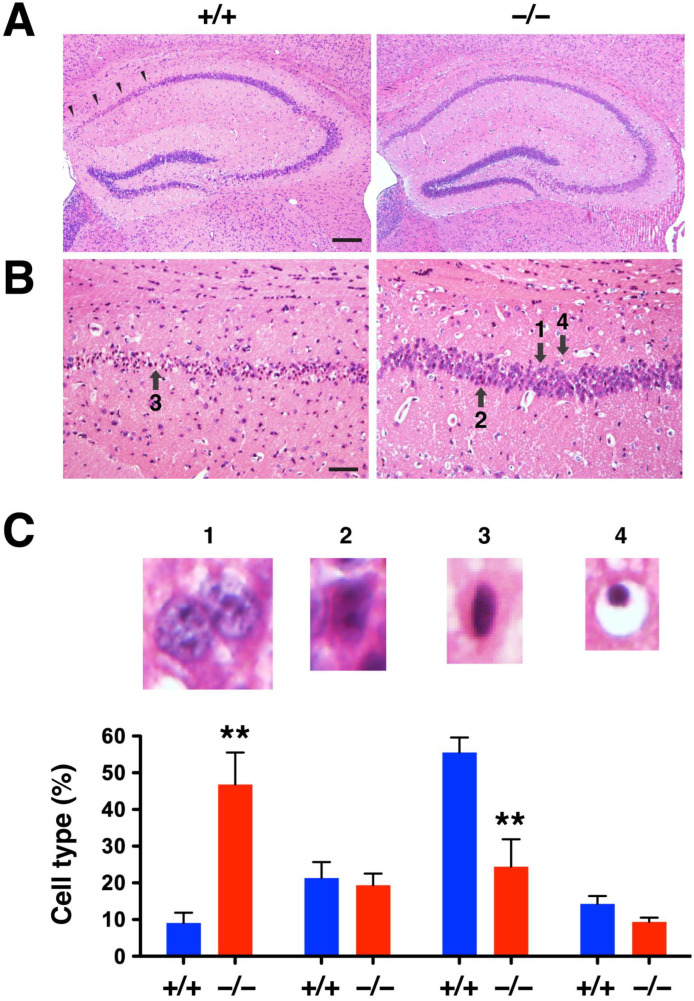
*DUSP6^−/−^* mice showed attenuated neuronal cell death after tGCI. (**A**) Representative images of H&E-stained brain sections from *DUSP6^+/+^* and *DUSP6^−/−^* mice 7 days after tGCI. Arrowheads indicate the area of damage in the hippocampal CA1 region. (**B**) Enlarged views of the hippocampal CA1 region showing degenerative neurons with eosinophilic (pink) cytoplasm and dark pyknotic (condensed) nuclei. Arrows indicate neurons in different stages of degeneration; 1: normal pyramidal neuron with a large and round nucleus; 2: degenerating neuron with ischemic eosinophilia in the cytoplasm; 3: degenerating neuron with eosinophilic cytoplasm and pyknotic nucleus; 4: degenerative neuron with pyknotic nucleus and empty cytoplasm. Scale bars, 200 μm (**A**) and 50 μm **(B)**. (**C**) Quantification of neuronal cell death in the hippocampal CA1 region of *DUSP6^+/+^* and *DUSP6^−/−^* mice 7 days after tGCI. The percentage of normal pyramidal neurons (1) was significantly higher in *DUSP6^−/−^* mice, whereas the percentage of degenerative neurons with eosinophilic cytoplasm and a pyknotic nucleus (3) was significantly lower in *DUSP6^−/−^* mice (*n* = 6 per group). ** *p* < 0.01 compared with *DUSP6^+/+^* mice (two-tailed, unpaired *t*-test).

Ischemia-induced neurodegeneration revealed by Fluoro-Jade C staining was evident in the hippocampal CA1 region of *DUSP6^+/+^* mice 7 days after tGCI (Figure 5A), but not in *DUSP6^−/−^* mice (Figure 5B). The degree of neurodegeneration was analyzed by counting the number of Fluoro-Jade C labeled neurons in the enlarged views of hippocampal CA1 regions of *DUSP6^+/+^* (Figure 5C) and *DUSP6^−/−^* (Figure 5D) mice. The difference in the number of Fluoro-Jade C-labeled neurons was statistically significant, with 69.5% reduction in the hippocampus of *DUSP6^−/−^* mice compared to *DUSP6^+/+^* mice (*p* = 0.048; Figure 5E). Provided that Fluoro-Jade C labeled not only degenerating neurons but also stressed neurons that may not eventually die [25,26,27], H&E staining was employed to confirm neurodegeneration after ischemia (Figure 6) [28]. Neuronal cell death revealed by H&E staining appeared in the hippocampal CA1 region of *DUSP6^+/+^* mice, but not *DUSP6^−/−^* mice, 7 days after tGCI (Figure 6A). Neurons in different stages of degeneration were identified and counted in the enlarged views of hippocampal CA1 regions of *DUSP6^+/+^* and *DUSP6^−/−^* mice (Figure 6B). The percentage of surviving neurons after tGCI was significantly higher in *DUSP6^−/−^* mice (46.9 ± 8.7 %) compared to *DUSP6^+/+^* mice (9.0 ± 2.8%; *p* = 0.002; Figure 6C). The percentage of degenerating neurons with eosinophilic cytoplasm and a pyknotic nucleus was significantly lower in *DUSP6^−/−^* mice (24.4 ± 7.5 %) compared to *DUSP6^+/+^* mice (55.5 ± 4.1 %; *p* = 0.0045; Figure 6C). Histological assessment of hippocampal injury after tGCI suggests that DUSP6 deficiency reduces neurodegeneration after tGCI.

### 2.4. DUSP6 Deficiency Enhanced Phosphorylation and Nuclear Translocation of Erk1/2 in Hippocampal CA1 Neurons

**Figure 7:**
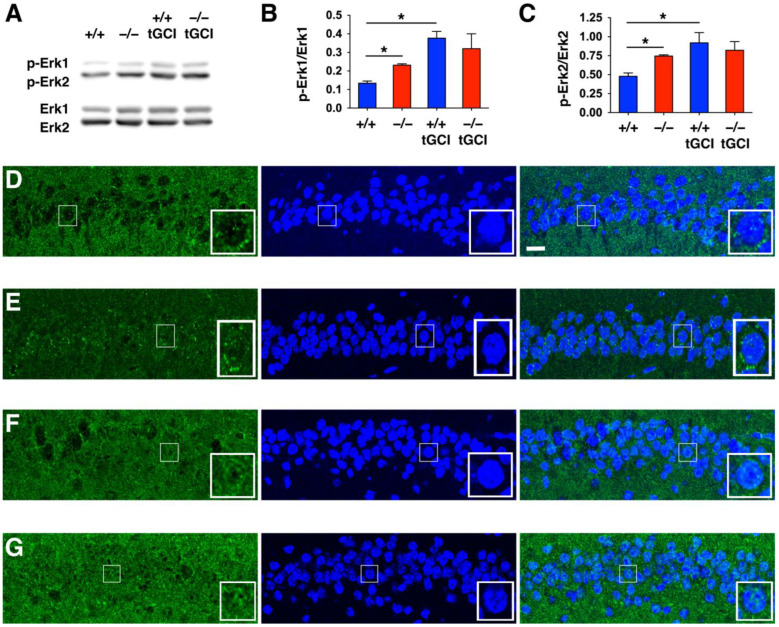
*DUSP6^−/−^* mice showed increased phosphorylation and nuclear translocation of Erk1/2 in the CA1 region of the hippocampus. (**A**) Representative Western blots showing the immunoreactivities of p-Erk1/2 and Erk1/2 in the hippocampal CA1 regions of naïve *DUSP6^+/+^* and *DUSP6^−/−^* mice, as well as in *DUSP6^+/+^* and *DUSP6^−/−^* mice at 24 h after tGCI. (**B**,**C**) P-Erk 1/2 and Erk1/2 protein bands were quantified by densitometry (*n* = 3 per group). There was a significant increase in p-Erk1/Erk1 and p-Erk2/Erk2 immunoreactivities in naïve *DUSP6^−/−^* mice compared with naïve *DUSP6^+/+^* mice (* *p* < 0.05, *t*-test). Significant induction of *p*-Erk1/Erk1 and p-Erk2/Erk2 immunoreactivities at 24 h after tGCI were detected in *DUSP6^+/+^*, but not in *DUSP6^−/−^* mice (* *p* < 0.05, *t*-test). Expression of Erk2 protein in the hippocampal CA1 regions of naïve *DUSP6^+/+^* (**D**) and *DUSP6^−/−^* (**E**) mice, as well as *DUSP6^+/+^* (**F**) and *DUSP6^−/−^* (**G**) mice at 24 h after tGCI was detected by immunofluorescence staining and confocal microscopy. Nuclei were stained with DAPI (blue). Representative neurons are amplified in the lower right corner of each panel. Scale bar, 10 μm.

DUSP6 is a protein phosphatase that specifically dephosphorylates Erk1/2 [12]. To assess the effect of DUSP6 deficiency on the phosphorylation of Erk1/2, we performed Western blotting of hippocampal lysates and found that the level of phosphorylation of Erk1/2 was enhanced in the hippocampus of naïve *DUSP6^−/−^* mice compared to naïve *DUSP6^+/+^* mice (Figure 7A–C). The phosphorylation of Erk1/2 was significantly increased at 24 h after tGCI in the hippocampus of *DUSP6^+/+^* mice, but not in *DUSP6^−/−^* mice (Figure 7A–C). Erk1/2 translocates to the nucleus once it is activated by phosphorylation [9,29]. To determine the subcellular location of Erk1/2 in hippocampal neurons, we performed immunofluorescence staining and found that Erk2 was detected in the cytoplasm of hippocampal CA1 neurons of naïve *DUSP6^+/+^* mice (Figure 7D). Interestingly, Erk2 was evenly distributed in both the nucleus and cytoplasm of hippocampal CA1 neurons of naïve *DUSP6^−/−^* mice (Figure 7E). Erk2 was detected in both the nucleus and cytoplasm of hippocampal CA1 neurons of *DUSP6^+/+^* (Figure 7F) and *DUSP6^−/−^* (Figure 7G) mice at 24 h after tGCI. More ERK2 was detected in the nucleus of *DUSP6^−/−^* mice (Figure 7G). These results suggest that DUSP6 inhibits the phosphorylation and nuclear translocation of Erk1/2 in hippocampal CA1 neurons.

## 3. Discussion

The mechanisms leading to ischemia-induced neurodegeneration in hippocampal CA1 neurons after cardiac arrest are unclear. We observed that DUSP6 mRNA and protein were transiently induced in hippocampal CA1 neurons after tGCI. *DUSP6^−/−^* mice showed attenuated neurodegeneration after tGCI, as assessed using Fluoro-Jade C and H&E staining. Ischemia-induced impairment of spatial learning and memory was reduced in *DUSP6^−/−^* mice, as assessed using the Barnes maze. The phosphorylation and nuclear translocation of Erk1/2 were enhanced in the hippocampal CA1 neurons of *DUSP6^−/−^* mice. This is the first study to reveal that DUSP6 mediates ischemia-induced neurodegeneration by regulating ERK1/2 signaling in response to tGCI.

ERK1/2 signaling plays a critical role in neurodegeneration; therefore, tight control of its magnitude and duration of expression, as well as its spatiotemporal activity, is required [8,9,10,11]. DUSP6 is a cytoplasmic DUSP that binds to, dephosphorylates, and inactivates Erk1/2 [12,13]. Erk1/2 is anchored in the cytoplasm by its interaction with DUSP6 under non-stimulated conditions [29]. In the absence of DUSP6, Erk1/2 phosphorylation was increased, and Erk2 was less sequestered in the cytoplasm of hippocampal CA1 neurons in *DUSP6^−/−^* mice (Figure 7). Activated Erk1/2 is known to translocate into the nucleus and phosphorylate its nuclear substrates; therefore, increased phosphorylation and nuclear translocation of Erk1/2 in *DUSP6^−/−^* mice may increase the access of Erk1/2 to nuclear substrates and provide neuroprotection against tGCI.

Ischemic preconditioning has gained attention as a potential neuroprotective therapy. It is defined as a state produced by brief episodes of sublethal ischemic stress that protects cells from a subsequent episode of potentially lethal ischemia [30,31]. ERK1/2 has been shown to play a protective role in ischemic preconditioning [8,30,31]. Preconditioning with mild tGCI for 3 min enhanced the phosphorylation of Erk1/2 in the CA1 region of the rat hippocampus [32]. Treatments with Erk1/2 inhibitor PD98059 [33,34] and U0126 [35] blocked the neuroprotective effects of preconditioning. The neuroprotective effects of DUSP6 deficiency may be due to upregulation of ERK1/2 signaling, thereby preconditioning the hippocampal CA1 neurons.

Previous studies using cell culture and pre-clinical rodent models have demonstrated that robust induction of ERK1/2 activity induces neuronal death, whereas mild elevation of ERK1/2 activity promotes cell survival [8,9,10,11]. We found that Erk1/2 phosphorylation was robustly induced after tGCI in *DUSP6^+/+^* mice when compared to naïve *DUSP6^+/+^* mice (Figure 7A–C). On the other hand, Erk1/2 phosphorylation was already elevated in naïve *DUSP6^−/−^* mice compared to naïve *DUSP6^+/+^* mice. Erk1/2 phosphorylation was not significantly induced in *DUSP6^−/−^* mice after tGCI compared to naïve *DUSP6^−/−^* mice. Our results suggest that the reduced magnitude of ERK1/2 induction after tGCI in *DUSP6^−/−^* mice may contribute to the attenuated neurodegeneration observed.

Hippocampal neurodegeneration is also observed in patients with ischemic stroke [36,37]. Ischemic stroke in the area of the middle cerebral artery causes neuronal damage in the infarcted core and induces neurodegeneration in the surrounding penumbra and non-ischemic regions that are synaptically connected with the primary lesion core, such as the ipsilateral hippocampus [38]. Current evidence suggests that post-stroke dementia rates are approximately 25–30% [36,37]. Therefore, specific DUSP6 inhibitors may prove useful in treating ischemia-induced neurodegeneration and dementia, benefitting both cardiac arrest and ischemic stroke patients.

Based on in vivo whole organism screens in transgenic zebrafish, (E/Z)-BCI hydrochloride (BCI) was identified as a specific, allosteric, cell-permeable, and small-molecule inhibitor for DUSP6 [39]. BCI binds with an allosteric pocket of DUSP6 which is adjacent to its phosphatase domain, inhibiting DUSP6 to dephosphorylate Erk1/2 through allosteric mechanisms. Treatments with BCI reduced DUSP6 phosphatase activity in vitro and increased Erk1/2 phosphorylation in zebrafish [39], mammalian cell culture [20,40], and mice [41]. Treatments with BCI increased cardiomyocyte proliferation and improved cardiac regeneration in zebrafish [42]. Intraperitoneal (i.p.) injections of BCI suppressed cancer cell proliferation, migration, and invasion in a mouse model of gastric cancer [41]. An analog of BCI (BCI-215) has been identified as a potent DUSP6 inhibitor with lower toxicity to zebrafish embryos and an endothelial cell line [43]. Evaluating the therapeutic potentials of BCI, BCI-215, and their derivatives in mitigating neurodegeneration induced by ischemia is of significant interest.

## 4. Materials and Methods

### 4.1. Transient Global Cerebral Ischemia (tGCI)

Male *DUSP6^+/+^* and *DUSP6^−/−^* mice, on a C57BL/6 background (Jackson Laboratory, Bar Harbor, ME, USA), between 2 and 5 months of age, were subjected to tGCI [28,44,45]. Mice were fasted overnight, with free access to water, before tGCI. On the day of surgery, blood glucose level was measured from tail-tip amputation using the ACCU-CHEK^®^ Aviva glucometer (Roche, Indianapolis, IN, USA). Mice with a blood glucose level between 100 and 150 mg/dL were selected for surgery. Mice were anesthetized with 4% isoflurane in a mixture of N_2_O/O_2_ (70%/30%), intubated, and maintained on 1.5% isoflurane using the MouseVent G500 Automatic Ventilator (Kent Scientific, Torrington, CT, USA). The tGCI was induced by a combination of hypotension and bilateral common carotid artery occlusion using microclips for 10 min. Mice developing seizures after tGCI were excluded from further study. All procedures were approved by the Institutional Animal Care and Use Committee (IACUC) at National Health Research Institutes (NHRI) in accordance with National Institutes of Health and ARRIVE (Animal Research: Reporting In Vivo Experiments) guidelines. The protocol numbers for this study are NHRI-IACUC-108019-M2-A-S01 and NHRI-IACUC-111062-A-S01.

In total, 94 mice were subjected to tGCI and randomly assigned into different groups of analysis. An amount of 15 mice were used for real-time RT-PCR (*n* = 3 per group). An amount of 12 mice were used for immunofluorescence staining to identify the expression pattern of DUSP6 in the hippocampus after tGCI (*n* = 3 per group). An amount of 28 mice were used for Barnes maze (8 naïve *DUSP6^+/+^*, 8 naïve *DUSP6^−/−^*, 6 *DUSP6^+/+^* after tGCI, and 6 *DUSP6^−/−^* mice after tGCI). An amount of 12 mice were used for Fluoro-Jade C staining (6 *DUSP6^+/+^* after tGCI and 6 *DUSP6^−/−^* mice after tGCI). An amount of 12 mice were used for H&E staining (6 *DUSP6^+/+^* after tGCI and 6 *DUSP6^−/−^* mice after tGCI). An amount of 12 mice were used for Western blotting (3 naïve *DUSP6^+/+^*, 3 naïve *DUSP6^−/−^*, 3 *DUSP6^+/+^* after tGCI, and 3 *DUSP6^−/−^* mice after tGCI). Three mice were used for immunofluorescence staining to identify the expression pattern of Erk2 in the hippocampus after tGCI.

### 4.2. Real-Time RT-PCR

Total RNA was extracted from the hippocampus at 4, 24, and 72 h, and 7 days after tGCI using TRIzol reagent (Invitrogen, Carlsbad, CA, USA) [46,47]. Isolated RNA was quantified using a NanoDrop 2000 Spectrophotometer (Thermo Fisher Scientific, Waltham, MA, USA) and reverse transcribed into cDNA using a RevertAid H Minus First Strand cDNA Synthesis Kit (Thermo) and a Veriti 96-Well Thermal Cycler (Thermo). Real-time PCR was performed using the StepOnePlus Real-Time PCR System (Thermo) and Luminaris Color HiGreen High ROX qPCR Master Mix (Thermo). The levels of DUSP6 mRNA at different time points after tGCI were determined using the 2^-ΔΔCT^ method, with mouse GAPDH mRNA as an internal control. The sequences of the primers used for DUSP6 were 5′-CTC GGA TCA CTG GAG CCA AAA C-3′ (forward) and 5′-TCT GCA TGA GGT ACG CCA CTG T-3′ (reverse), and for GAPDH, were 5′-CCA TTT GCA GTG GCA AAG-3′ (forward) and 5′-CAC CCC ATT TGA TGT TAG TG-3′ (reverse).

### 4.3. Immunofluorescence Staining

Mice were anesthetized deeply with 4% isoflurane in N_2_O/O_2_ (70%/30%) at 4, 24, and 48 h after tGCI, and transcardially perfused with saline [44,46,47,48]. Mouse brains were isolated, sectioned using a brain matrix (Braintree Scientific, Braintree, MA, USA) to generate 1 mm-thick coronal sections, and fixed overnight in 10% neutral buffered Formalin solution (Sigma-Aldrich, St. Louis, MO, USA, Cat# HT501128) for paraffin embedding. Paraffin-embedded brain sections (5 μm) containing the hippocampus (−1.67 to −1.91 mm caudal to bregma) were prepared, deparaffinized, and rehydrated. Antigen retrieval was performed in 1X Trilogy buffer (Cell Marque, Rocklin, CA, USA, Cat#920P-06) using a pressure cooker (Cuisinart, Stamford, CT, USA, Cat# CPC-600) for 10 min at low pressure [49]. Sections were incubated in blocking buffer containing 1X PBS and 5% normal donkey serum (Jackson ImmunoResearch, West Grove, PA, USA, Cat# 017-000-121) for 1 h at room temperature and incubated overnight at 4 °C with rabbit anti-DUSP6 (1:200, Abcam, Cambridge, UK, Cat# ab76310), mouse anti-MAP2 (1:400, Millipore, Burlington, MA, USA, Cat# MAB3418), goat anti-GFAP (1:1000, Abcam, Cat# ab53554), or goat anti-Iba1 (1:200, Abcam, Cat# ab48004) antibodies in antibody dilution buffer containing 1X PBS and 2% normal donkey serum. After washing, brain sections were stained with Alexa Fluor conjugated secondary antibodies (1:200, Jackson ImmunoResearch, West Grove, PA, USA) in antibody dilution buffer containing 1X PBS and 2% normal donkey serum, and mounted in media containing 4′, 6-diamidino-2-phenylindole (DAPI; Vector Laboratories, Burlingame, CA, USA). Images were acquired using a Leica TCS SP5 II confocal laser scanning microscope.

### 4.4. Barnes Maze

Spatial memory acquisition and retention by *DUSP6^+/+^* and *DUSP6^−/−^* mice before and after tGCI were assessed using the Barnes maze [45,50]. The Barnes maze, a circular platform (92 cm in diameter) with 20 evenly spaced holes (5 cm in diameter) along the perimeter, was placed in the center of a testing room that had visual cues attached to the wall. Of the 20 holes, 1 was designated as the escape target by placement of a black acrylic box (27 × 9 × 6 cm) with a stepped-down ramp under the hole. Mice were trained to escape into the target hole in 4 trials (3 min each) per day for 4 consecutive days. On day 5, a probe trial was conducted with the escape box removed from the maze. Mice were allowed to explore the maze for 90 sec. Path tracings and the number of nose pokes at the target hole during the probe trial were recorded with a video camera and analyzed using EthoVision software v. 7.0 (Noldus Information Technology, Leesburg, VA, USA).

### 4.5. Fluoro-Jade C and H&E Staining

Paraffin embedded brain sections (5 μm) containing the hippocampus (−1.67 to −1.91 mm caudal to bregma) were prepared 7 days after tGCI and stained with hematoxylin and eosin (H&E) or Fluoro-Jade C following the manufacturer’s protocol (Histo-Chem, Jefferson, AR, USA) [44,45]. Neurons with positive Fluoro-Jade C staining in the hippocampus were counted by an investigator blinded to the treatment conditions. Neurons that degenerate after tGCI exhibit eosinophilic (acidophilic, pink) cytoplasm and dark pyknotic (condensed) nuclei after H&E staining [28,45,51]. Healthy neurons with a large and round nucleus (type 1), as well as degenerating neurons with eosinophilia in the cytoplasm (type 2), eosinophilic cytoplasm and a pyknotic nucleus (type 3), and a pyknotic nucleus and empty cytoplasm (type 4), were counted by an investigator blinded to the treatment conditions. About 300–400 degenerating neurons were counted for each hippocampus. Neurons in both right and left hippocampus were counted and combined. The numbers of type 1, 2, 3, and 4 neurons were divided by the total number of counted neurons (about 600–800 neurons) to calculate the percentages of different types of neurons.

### 4.6. Western Blotting

The CA1 region of hippocampus was dissected from naïve *DUSP6^+/+^* and *DUSP6^−/−^* mice, as well as from *DUSP6^+/+^* and *DUSP6^−/−^* mice 24 h after tGCI. Tissues were placed in a Teflon-glass homogenizer and homogenized in ice-cold RIPA buffer containing 5 mM EDTA, 1 mM PMSF, 10 nM Pepstatin A, and 1X Halt Protease and Phosphatase inhibitors. The homogenate was centrifuged at 20,000 X g for 10 min at 4 °C. The supernatant was collected for Western blotting using rabbit anti-phospho-p44/42 MAPK (Erk1/2; Thr202/Tyr204) (1:1000; Cell Signaling, Danvers, MA, USA, Cat# 4370) and anti-p44/42 MAPK (Erk1/2) (1:1000; Cell Signaling, Cat# 9102).

### 4.7. Statistics

Quantitative data were expressed as means ± SEM and analyzed by *t*-test, one-way ANOVA, two-way ANOVA, and Tukey’s post hoc test using Prism 9 (GraphPad, La Jolla, CA, USA). A statistically significant difference was defined as *p* < 0.05.

## Data Availability

The raw data supporting the conclusions of this article will be made available by the authors, without undue reservation.

## Abbreviations

Dual-specific phosphatase-6 (DUSP6); mitogen-activated protein kinase phosphatase-3 (MKP3); extracellular signal-regulated kinase 1/2 (ERK1/2); mitogen-activated protein kinase (MAPK); transient global cerebral ischemia (tGCI); cardiopulmonary resuscitation (CPR); cornu ammonis 1 (CA1); dentate gyrus (DG); glyceraldehyde-3-phosphate dehydrogenase (GAPDH); 4′, 6-diamidino-2-phenylindole (DAPI); RIPA (radio-immunoprecipitation assay) buffer; immunohistochemistry (IHC); ethylenediaminetetraacetic acid (EDTA); phenylmethanesulfonyl fluoride (PMSF); microtubule-associated protein 2 (MAP2); glial fibrillary acidic protein (GFAP); ionized calcium-binding adapter molecule 1 (Iba1); hematoxylin and eosin (H&E); Fluoro-Jade C (FJC); analysis of variance (ANOVA); (E/Z)-BCI hydrochloride (BCI); (E/Z)-BCI hydrochloride 215 (BCI-215).
